# Goserelin (Zoladex) in premenopausal advanced breast cancer: duration of response and survival.

**DOI:** 10.1038/bjc.1990.397

**Published:** 1990-11

**Authors:** A. R. Dixon, J. F. Robertson, L. Jackson, R. I. Nicholson, K. J. Walker, R. W. Blamey

**Affiliations:** Department of Surgery, City Hospital, Nottingham, UK.

## Abstract

In premenopausal women with advanced breast cancer the luteinising hormone-releasing hormone agonist goserelin (Zoladex, ICI plc) will produce serum levels of oestradiol equivalent to those following surgical oophorectomy or the menopause. This paper reports our further experience of using this drug in 75 premenopausal patients with advanced breast cancer. In addition to response rates, duration of response is reported. An objective response was seen in 25 patients (33%), the median duration of which was in excess of 15 months. Seven patients (9%) showed a complete response to therapy; median duration greater than 37 months. There was no significant difference in time to disease progression (Lee-Desu statistic 18.26, 1 d.f., P = 0.43) and probability of survival (Lee-Desu statistic 3.41, 1 d.f., P = 0.07) between those patients assessed as having either static disease, or those showing a partial response at six months. Response to therapy correlates significantly with the oestrogen receptor status of the primary tumour (X2 = 20.59, 6 d.f., P less than 0.005). The modest side-effects, ease of administration and reversibility make this approach to therapy very attractive. This is to be remembered in that 53% of patients had disease progression whilst receiving goserelin. These patients thus avoided the unnecessary and irreversible morbidity associated with surgical oophorectomy. With the proven efficacy and minimal morbidity associated with goserelin we believe there is no current role for surgical oophorectomy in the management of premenopausal patients with advanced breast cancer.


Br. J. Cancer (1990), 62, 868-870 © Macmillan Press Ltd., 1990

### Goserelin (Zoladex) in premenopausal advanced breast cancer: duration of response and survival

A.R. Dixon[1], J.F.R. Robertson[1], L. Jackson[1], R.I. Nicholson[2], K.J. Walker[2] & R.W. Blamey[1]

[1]Department of Surgery, City Hospital, Hucknall Road, Nottingham NG5 6JE; and [2]Tenovus Institute for Cancer Research, University of Wales College of Medicine, Cardiff CF4 4XX, UK.

**Summary** In premenopausal women with advanced breast cancer the luteinising hormone-releasing hormone agonist goserelin (Zoladex, ICI plc) will produce serum levels of oestradiol equivalent to those following surgical oophorectomy or the menopause. This paper reports our further experience of using this drug in 75 premenopausal patients with advanced breast cancer. In addition to response rates, duration of response is reported. An objective response was seen in 25 patients (33%), the median duration of which was in excess of 15 months. Seven patients (9%) showed a complete response to therapy; median duration >37 months. There was no significant difference in time to disease progression (Lee-Desu statistic 18.26, 1 d.f., $P = 0.43$) and probability of survival (Lee-Desu statistic 3.41, 1 d.f., $P = 0.07$) between those patients assessed as having either static disease, or those showing a partial response at six months. Response to therapy correlates significantly with the oestrogen receptor status of the primary tumour ($\chi^2 = 20.59$, 6 d.f., $P < 0.005$). The modest side-effects, ease of administration and reversibility make this approach to therapy very attractive. This is to be remembered in that 53% of patients had disease progression whilst receiving goserelin. These patients thus avoided the unnecessary and irreversible morbidity associated with surgical oophorectomy. With the proven efficacy and minimal morbidity associated with goserelin we believe there is no current role for surgical oophorectomy in the management of premenopausal patients with advanced breast cancer.

The endocrine effects and clinical efficacy of the luteinising hormone-releasing hormone (LH-RH) agonist goserelin (Zoladex, ICI plc) as initial hormone therapy for premenopausal advanced breast cancer patients have been previously reported (Nicholson *et al.*, 1984, 1985; Williams *et al.*, 1986; Walker *et al.*, 1986). In a study of 53 premenopausal patients we reported a response rate to goserelin of 31%; comparable to our previous experience of surgical oophorectomy (Williams *et al.*, 1986; Buchanan *et al.*, 1986). Recently we have described our experience of combining goserelin with the anti-oestrogen tamoxifen (Robertson *et al.*, 1989*a*; Walker *et al.*, 1989). Slightly lower levels of serum oestradiol were recorded in those patients receiving combination therapy, along with significant reductions in FSH as compared with goserelin alone. An international multicentre trial is currently underway to determine any clinical advantage of such a combination.

The current paper updates our experience of treating 75 premenopausal advanced breast cancer patients with monthly depot injections of goserelin 3.6 mg; particular address has been given to both the duration of response and survival probability.

### Patients and methods

Seventy-five premenopausal patients with histologically proven advanced breast cancer have been treated by the administration of the gonadotrophin releasing hormone agonist goserelin, subcutaneous implantation of a 3.6 mg depot preparation at 28 day intervals. No patient had received previous endocrine or cytotoxic therapies: all gave written informed consent to the administration of the drug. The median age of the patients on commencing therapy was 44 years (range 31-55), with the sites of disease as shown in Table I. The major sites of metastatic disease in 54 patients were: bone, 22 patients; pulmonary, 14 patients; bone and pulmonary, 12 patients; visceral, six patients.

Initial examination included a full clinical examination with documentation of all measurable disease and photography where appropriate. A limited skeletal survey was obtained in all patients. CT scans, isotope bone scans and liver sonography were performed when clinically indicated. Routine haematological and biochemical estimations were also performed. Tumour steroid hormone receptor status was known in 60 patients; the oestrogen receptor assays having been performed by the Tenovus Institute, Cardiff using the commercially available ER-enzyme immuno assay (Abbot ER-Eia, monoclonal). Oestrogen receptor was considered positive when a value greater than 5 fmol $mg^{-1}$ cytosol protein was obtained (Nicholson *et al.*, 1981).

Patients were assessed for response according to UICC criteria (Hayward *et al.*, 1977). The British Breast Group recommendation that the minimum duration of remission be six months was also adhered to (British Breast Group, 1974).

**Table I** Sites of disease in 75 patients receiving monthly, depot, subcutaneous goserelin

| | *No. of patients* |
|---|---|
| Locally advanced primary | 15 |
| Locoregional recurrence | 6 |
| Locoregional recurrence with metastases | 16 |
| Metastatic disease | 38 |

### Statistical methods

Actuarial survival analysis was performed using the statistical package SPSSX-21 (SPSS, 1986) life table analysis which calculates Gehan's generalised Wilcoxon rank test for censored data (Lee & Desu, 1972).

### Results

Twenty-five of the 75 patients (33%) in whom disease was assessable using the strict clinical criteria of the UICC (Hayward *et al.*, 1977) and the BBG (British Breast Group, 1974) were classified as having shown an objective response to therapy of at least 6 months duration. A complete response (CR) was seen in seven patients (9%), the median duration of which was in excess of 37 months. Three of these responders had stage III disease, while four had locoregional recurrence; three of whom also had metastases (two pulmonary, one bone). Static disease (SD) was seen in a further 11 patients (15%), whilst the remaining 39 patients (52%) progressed (PD) within 6 months of starting goserelin. Duration of response is recorded in Table II. The probability of

Correspondence: A.R. Dixon.
Received 16 February 1990; and in revised form 18 May 1990.

**Figure 1** Probability of disease progression for patients receiving goserelin.

disease progression in response to therapy is summarised in Figure 1. There is no significant difference in the time to disease progression between those patients assessed as having shown a partial response (PR) to goserelin at 6 months, and those that have statis disease (Lee-Desu statistic 0.63, 1 d.f., $P = 0.43$); survival does not significantly differ between these two groups (Lee-Desu statistic 3.41, 1 d.f., $P = 0.07$). Patients showing a complete response had a significantly increased time interval to disease progression as compared to those that showed a partial response (Lee-Desu statistic 6.69, 1 d.f., $P = 0.009$). The side-effects of goserelin included amenorrhoea, hot flushes, vaginal dryness, and occasional nausea.

Primary tumour oestrogen receptor (ER) status was available in 60 patients (80%); 32 patients were ER positive, and 28 ER negative (see Table III). Nineteen of the 24 patients responding to goserelin were ER positive, while three had unknown receptor status. Of the seven patients that showed a complete response, six had ER positive primary tumours. Twenty-eight patients had tumours that were ER negative; 21 of these had progressive disease. The oestrogen receptor status of the primary tumour correlates significantly with the prediction of response to goserelin ($\chi^2 = 20.59$, 6 d.f., $P < 0.005$).

### Discussion

Surgical oophorectomy has become the mainstay of treatment for premenopausal patients with advanced breast cancer since it was first introduced by Beatson at the end of the last century (Beatson, 1896). This surgical approach suffers many disadvantages in that treatment is palliative and the majority of patients will not respond, thus exposing many to unnecessary and irreversible morbidity (Kennedy *et al.*, 1964).

**Table II** Duration of response (months)

| *Response* | *Number* | *Duration* |
|---|---|---|
| CR | 7 (9%) | 17, 36, 36 +, 37, 38, 48 +, 52 + (median 37 +) |
| PR | 18 (24%) | 7, 7 +, 8, 8 +, 9, 9, 9, 11, 14 +, 14, 14, 15 +, 16 +, 16, 18, 18 +, 22 +, 36 (median 14 +) |
| SD | 11 (15%) | 6, 7, 8, 9, 9, 10 +, 10, 16 +, 18, 37, 45 +, (median 10 +) |
| PD | 39 (52%) | |

**Table III** Response to goserelin versus ER status of the primary tumour

| | *No. of patients* | | |
|---|---|---|---|
| | *ER pos.* | *ER neg.* | *Unknown* |
| Complete response | 6 | 1 | – |
| Partial response | 13 | 2 | 3 |
| Static disease | 5 | 4 | 2 |
| Progressive disease | 8 | 21 | 10 |

$\chi^2 = 20.59$, 6 d.f., $P < 0.005$.

Administration of the LH-RH agonist goserelin to premenopausal advanced breast cancer patients produces a rapid desensitisation of the pituitary gland to endogenous LH-RH, with resultant falls in the circulating levels of LH and FSH (Nicholson *et al.*, 1984, 1985; Williams *et al.*, 1986; Walker *et al.*, 1986). Castrate levels of oestradiol and progesterone are produced within 3–4 weeks although a small group of patients will shown recurrent suppressed peaks of oestradiol (Williams *et al.*, 1986). This ability to reduce serum oestradiol is not influenced by either the patients age or weight (Nicholson & Walker, 1989). A theoretical limiting factor to treatment with LH-RH agonists, as with radiation castration is their inability to immediately suppress ovarian activity; surgical oophorectomy produces castrate levels of oestradiol within 2 to 7 days (Vermeulen, 1976; Beksac *et al.*, 1983). Despite these theoretical shortcomings we reported a response to goserelin of 31% in a phase I study of 53 premenopausal patients (Williams *et al.*, 1986), a value that is comparable to our previous experiences using surgical oophorectomy (Buchanan *et al.*, 1986). When we examined the results of 23 assessable patients who had received a combination of goserelin and the antioestrogen tamoxifen we reported a 22% response, with a further 22% of patients showing static disease (Robertson *et al.*, 1989*a*).

It is apparent from this study of 75 patients that LH-RH agonists are capable of achieving a significant objective response of worthwhile duration in premenopausal advanced breast cancer patients. We report an objective response rate of 33%, with a median duration of response of 15 months (range 7–52). Seven patients (9%) were classified as having had a complete response, the median duration of which was in excess of 37 months. A further 11 patients (15%) had stable disease of at least 6 months duration (median duration 10 months). This group of patients have a similar survival to those that show responsive disease to at 6 months; this concords to our findings in patients treated with megesterol (Robertson *et al.*, 1989*b*). The remaining 39 patients (52%) had disease progression within 6 months of starting treatment.

It would appear that the presence of the oestrogen receptor in the primary tumour may be predictive of a response to medical oophorectomy using goserelin. This is in accordance to our previous findings (Williams *et al.*, 1986).

Goserelin is easily administered and produces an effective but reversible castration. Objective remissions of worthwhile duration are seen in a third of patients, comparable to surgical oophorectomy but without its potential and irreversible morbidity, psychological traumas and surgical risks. This is particularly important in that 50% of patients will show no response to the ovarian suppression. Monthly administration also ensures a high patient compliance.

**Figure 2** Probability of survival in patients receiving goserelin.

### References

BEATSON, G.T. (1896). On the treatment of inoperable cases of carcinoma of the mamma; suggestions for a new method of treatment with illustrative cases. *Lancet*, **ii**, 104.

BEKSAC, M.S., KISNISCI, H.A., CAKAR, A.N. & BEKSAC, M. (1983). The endocrinological evaluation of bilateral and unilateral oophorectomy in premenopausal women. *Int. J. Fertil.*, **28**, 219.

BRITISH BREAST GROUP (1974). Assessment of response to treatment in advanced breast cancer. *Lancet*, **ii**, 38.

BUCHANAN, R.B., BLAMEY, R.W., DURRANT, K.R. & 6 others (1986). A randomised comparison of tamoxifen with surgical oophorectomy in premenopausal patients with advanced breast cancer. *J. Clin. Oncol.*, **4**, 1326.

HAYWARD, J.L., CARBONE, P.P., HEUSON, J.C., KUMAOKA, S., SEGALOF, A. & RUBENS, R.D. (1977). Assessment of response to therapy in advanced breast cancer. A project of the International Union Against Cancer, Geneva, Switzerland. *Cancer*, **39**, 1289.

KENNEDY, B.J., MIELKE, P.W. & FORTUNY, I.E. (1964). Therapeutic castration verses prophylactic castration in breast cancer. *Surg. Gynecol. Obstet.*, **118**, 524.

LEE, E.T. & DESU, M.M. (1972). A computer program for comparing k samples with right censored data. *Comp. Prog. Biomed.*, **2**, 315.

NICHOLSON, R., CAMPBELL, F.C., BLAMEY, R.W., ELSTON, C.W., GEORGE, D. & GRIFFITHS, K. (1981). Steroid receptors in early breast cancer: value in prognosis. *J. Steroid Biochem.*, **15**, 193.

NICHOLSON, R.I., WALKER, K.J., TURKES, A. & 6 others (1984). Therapeutic significance and the mechanisms of action of the LH-RH agonist ICI 118630 in breast and prostatic cancer. *J. Steroid Biochem.*, **20**, 129.

NICHOLSON, R.I., WALKER, K.J., TURKES, A. & 4 others (1985). Endocrinological and clinical aspects of LH-RH action (ICI 118630) in hormone dependent breast cancer. *J. Steroid Biochem.*, **23**, 843.

NICHOLSON, R.I. & WALKER, K.J. (1989). Use of LH-RH agonists in the treatment of breast disease. *Proc. R. Soc. Edin. B.*, **95**, 271.

ROBERTSON, J.F.R., WALKER, K.J., NICHOLSON, R.I. & BLAMEY, R.W. (1989*a*). The combined endocrine effects of LH-RH agonist (Zoladex) and tamoxifen (Nolvadex) therapy in premenopausal women with breast cancer. *Br. J. Surg.*, **76**, 1262.

ROBERTSON, J.F.R., WILLIAMS, M.R., TODD, J., NICHOLSON, R.I., MORGAN, D.A.L. & BLAMEY, R.W. (1989*b*). Factors predicting the response of patients with advanced breast cancer to endocrine (Megace) therapy. *Eur. J. Clin. Oncol.*, **25**, 469.

SPSS (1986). *SPSS$^X$ User's Guide*. McGraw-Hill: New York.

VERMEULEN, A. (1976). The hormonal activity of the post menopausal ovary. *J. Clin. Endocrinol. Metab.*, **42**, 247.

WALKER, K.J., TURKES, A., WILLIAMS, M.R., BLAMEY, R.W. & NICHOLSON, R.I. (1986). Preliminary endocrinological evaluation of a sustained release formulation of the LH-releasing hormone agonist D-Ser (Bu)$^6$ Axgly$^{10}$ LH-RH in premenopausal women with advanced breast cancer. *J. Endocrinol.*, **111**, 349.

WALKER, K.J., WALKER, R.F., TURKES, A. & 4 others (1989). Endocrine effects of combination antioestrogen and LH-RH agonist therapy in premenopausal patients with advanced breast cancer. *Eur. J. Cancer Clin. Oncol.*, **25**, 651.

WILLIAMS, M.R., WALKER, K.J., TURKES, A., BLAMEY, R.W. & NICHOLSON, R.I. (1986). The use of an LH-RH agonist (ICI 118630, Zoladex) in advanced premenopausal breast cancer. *Br. J. Cancer*, **53**, 629.


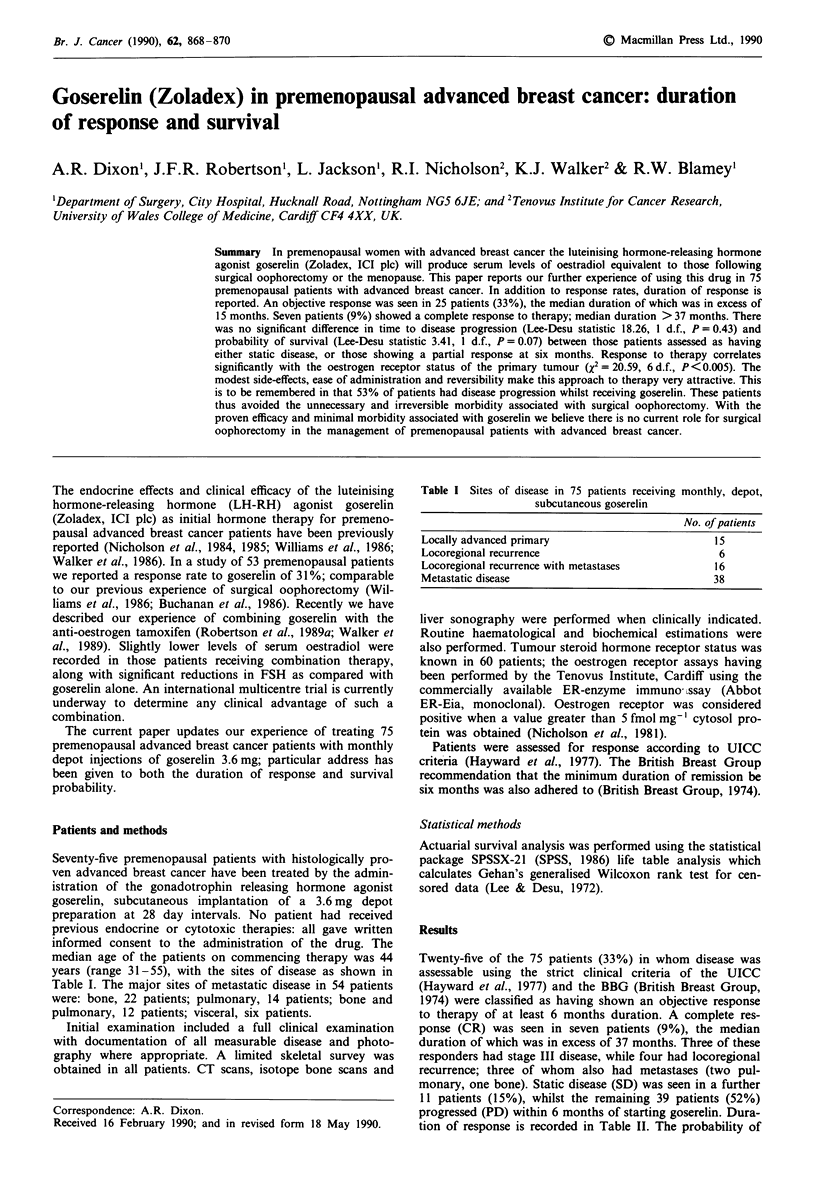

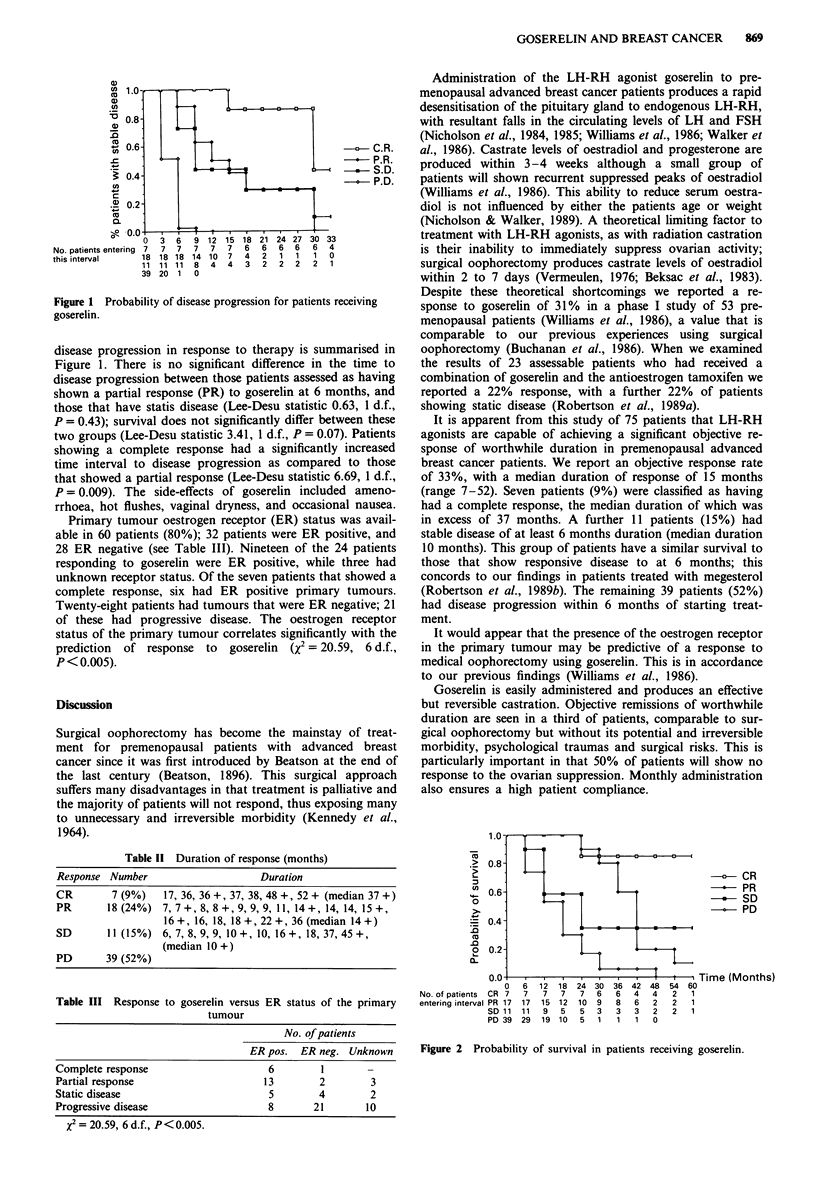

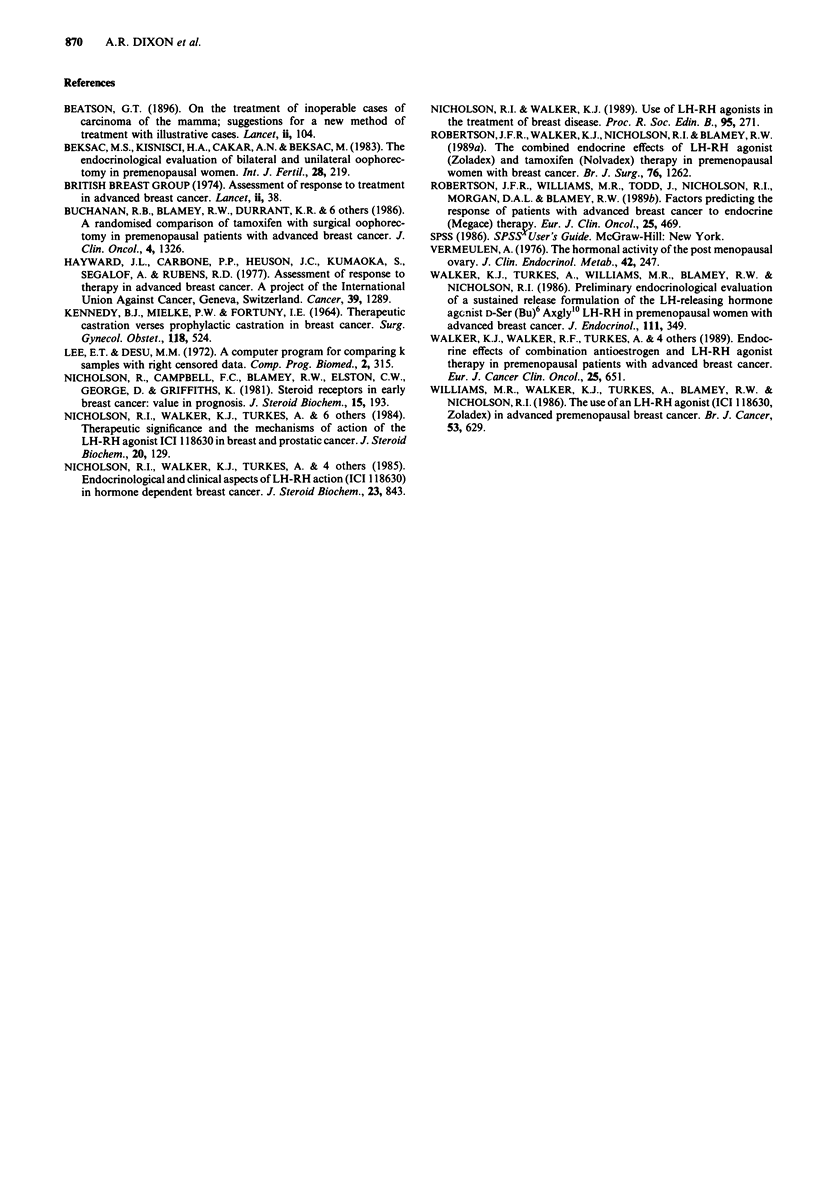


## References

[OCR_00329] Beksaç M. S., Kişnişci H. A., Cakar A. N., Beksaç M. (1983). The endocrinological evaluation of bilateral and unilateral oophorectomy in premenopausal women.. Int J Fertil.

[OCR_00338] Buchanan R. B., Blamey R. W., Durrant K. R., Howell A., Paterson A. G., Preece P. E., Smith D. C., Williams C. J., Wilson R. G. (1986). A randomized comparison of tamoxifen with surgical oophorectomy in premenopausal patients with advanced breast cancer.. J Clin Oncol.

[OCR_00344] Hayward J. L., Carbone P. P., Heuson J. C., Kumaoka S., Segaloff A., Rubens R. D. (1977). Assessment of response to therapy in advanced breast cancer: a project of the Programme on Clinical Oncology of the International Union Against Cancer, Geneva, Switzerland.. Cancer.

[OCR_00350] KENNEDY B. J., MIELKE P. W., FORTUNY I. E. (1964). THERAPEUTIC CASTRATION VERSUS PROPHYLACTIC CASTRATION IN BREAST CANCER.. Surg Gynecol Obstet.

[OCR_00355] Lee E. T., Desu M. M. (1972). A computer program for comparing K samples with right-censored data.. Comput Programs Biomed.

[OCR_00359] Nicholson R. I., Campbell F. C., Blamey R. W., Elston C. W., George D., Griffiths K. (1981). Steroid receptors in early breast cancer: value in prognosis.. J Steroid Biochem.

[OCR_00370] Nicholson R. I., Walker K. J., Turkes A., Dyas J., Plowman P. N., Williams M., Blamey R. W. (1985). Endocrinological and clinical aspects of LHRH action (ICI 118630) in hormone dependent breast cancer.. J Steroid Biochem.

[OCR_00364] Nicholson R. I., Walker K. J., Turkes A., Turkes A. O., Dyas J., Blamey R. W., Campbell F. C., Robinson M. R., Griffiths K. (1984). Therapeutic significance and the mechanism of action of the LH-RH agonist ICI 118630 in breast and prostate cancer.. J Steroid Biochem.

[OCR_00379] Robertson J. F., Walker K. J., Nicholson R. I., Blamey R. W. (1989). Combined endocrine effects of LHRH agonist (Zoladex) and tamoxifen (Nolvadex) therapy in premenopausal women with breast cancer.. Br J Surg.

[OCR_00385] Robertson J. F., Williams M. R., Todd J., Nicholson R. I., Morgan D. A., Blamey R. W. (1989). Factors predicting the response of patients with advanced breast cancer to endocrine (Megace) therapy.. Eur J Cancer Clin Oncol.

[OCR_00393] Vermeulen A. (1976). The hormonal activity of the postmenopausal ovary.. J Clin Endocrinol Metab.

[OCR_00397] Walker K. J., Turkes A., Williams M. R., Blamey R. W., Nicholson R. I. (1986). Preliminary endocrinological evaluation of a sustained-release formulation of the LH-releasing hormone agonist D-Ser(But)6Azgly10LHRH in premenopausal women with advanced breast cancer.. J Endocrinol.

[OCR_00404] Walker K. J., Walker R. F., Turkes A., Robertson J. F., Blamey R. W., Griffiths K., Nicholson R. I. (1989). Endocrine effects of combination antioestrogen and LH-RH agonist therapy in premenopausal patients with advanced breast cancer.. Eur J Cancer Clin Oncol.

[OCR_00410] Williams M. R., Walker K. J., Turkes A., Blamey R. W., Nicholson R. I. (1986). The use of an LH-RH agonist (ICI 118630, Zoladex) in advanced premenopausal breast cancer.. Br J Cancer.

